# Optimized MALDI TOF Mass Spectrometry Identification of *Francisella tularensis* Subsp. holarctica

**DOI:** 10.3390/microorganisms8081143

**Published:** 2020-07-28

**Authors:** Sofiane Regoui, Aurélie Hennebique, Thomas Girard, Sandrine Boisset, Yvan Caspar, Max Maurin

**Affiliations:** 1 Centre National de Référence des Francisella, Institut de Biologie et de Pathologie, Centre Hospitalier Universitaire Grenoble Alpes, 38043 Grenoble, France; regoui.sofiane@gmail.com (S.R.); AHennebique@chu-grenoble.fr (A.H.); TGirard1@chu-grenoble.fr (T.G.); SBoisset@chu-grenoble.fr (S.B.); YCaspar@chu-grenoble.fr (Y.C.); 2 Université Grenoble Alpes, Centre National de la Recherche Scientifique, Grenoble INP, CHU Grenoble Alpes, TIMC-IMAG, 38000 Grenoble, France

**Keywords:** *Francisella tularensis*, tularemia, identification, MALDI TOF mass spectrometry

## Abstract

*Francisella tularensis* is a tier 1 agent causing the zoonosis tularemia. This highly infectious Gram-negative bacterium is occasionally isolated from human samples (especially blood samples) in routine clinical microbiology laboratories. A rapid and accurate method for identifying this pathogen is needed in order to optimize the infected patient’s healthcare management and prevent contamination of the laboratory personnel. MALDI TOF mass spectrometry has become the gold standard for the rapid identification of most human pathogens. However, *F. tularensis* identification using such technology and commercially available databases is currently considered unreliable. Real-time PCR-based methods for rapid detection and accurate identification of *F. tularensis* are not available in many laboratories. As a national reference center for tularemia, we developed a MALDI TOF database allowing accurate identification of the species *F. tularensis* and its differentiation from the closely related neighbor species *F. tularensis* subsp. *novicida* and *F. philomiragia*. The sensitivity and specificity of this database were validated by testing 71 *F. tularensis strains* and 165 strains from 63 species not belonging to the *Francisella* genus. We obtained accurate identification at the species level and differentiation of all the tested bacterial strains. In particular, *F. tularensis* could be accurately differentiated from other small Gram-negative bacilli occasionally isolated from human samples, including species of the HACEK group and *Brucella melitensis*.

## 1. Introduction

Tularemia is a zoonosis classically caused by *Francisella tularensis* [1]. However, *F. tularensis* contains four subspecies, of which only two are etiological agents of tularemia: *F. tularensis* subsp. *tularensis* (type A) in North America and *F. tularensis* subsp. *holarctica* in the whole northern hemisphere [1]. Type A strains are classified as category A of the CDC’s biological threat agents (Centers for Diseases Control and Prevention, Atlanta, USA) [2,3]. Type B strains have recently been detected in Southern Australia [4]. *F. tularensis* subsp. *mediasiatica* is restricted to Central Asia and Russia but has never been isolated in humans [5]. *F. tularensis* subsp. *novicida* is a waterborne bacterium occasionally associated with human infections [6]. Human tularemia cases are most often sporadic. However, tularemia outbreaks have been reported in the last two decades in many countries, including Scandinavia, Turkey, and Spain [7,8,9,10]. The incidence of tularemia in France is evaluated as between 0.08 and 0.25 cases per 10^5^ inhabitants, which corresponds to 50 to 150 annual cases declared to Santé Publique France (the French Institute for Disease surveillance) [11].

Following a short incubation period (3–5 days on average), six clinical forms of tularemia are classically recognized [1,12,13]. The ulceroglandular and glandular forms correspond to regional lymphadenopathy, with or without a skin inoculation lesion, respectively. The oculoglandular and pharyngeal forms correspond, respectively, to conjunctivitis or pharyngitis, with regional lymphadenopathy. The pneumonic (pneumonia or pleuropneumonia) and typhoidal (typhoid-like) forms are systemic, occasionally fatal diseases [3].

Tularemia diagnosis is mainly based on serology [1,12,13]. Several techniques may be used for specific antibody detection, including the microagglutination test (MAT), the immunofluorescence assay (IFA), and ELISA tests. Significant titers of specific antibodies are usually detected 2–3 weeks after symptom onset. The isolation of *F. tularensis* from clinical samples remains the gold standard. However, a positive culture is obtained in approximately 10% of infected patients only [12,13]. In many instances, this bacterium is isolated from blood culture samples in patients with fever and unspecific clinical symptoms. Finally, the detection of *F. tularensis* DNA in clinical samples is also diagnostic. The WHO considers as proven tularemia cases patients with compatible clinical and epidemiological findings and one of the following criteria: a *F. tularensis* positive culture or PCR, a seroconversion, or a fourfold or higher rise in antibody titers between two serum samples collected two weeks apart or more [14]. A positive serological test in a compatible clinical and epidemiological context is considered a probable tularemia case.

In patients with a positive *F. tularensis* culture, rapid identification of this highly infectious pathogen is warranted in order to optimize the patient’s management and prevent contamination of the laboratory personnel [15]. This bacterium is poorly reactive in most biochemical tests, making its phenotypic identification unreliable [16,17]. Monoclonal antibodies have been developed to detect *Francisella* bacteria, but antigenic cross-reactions between *Francisella* species are frequent [18,19]. Thus, conventional identification techniques are not suitable for the rapid identification of *Francisella* species. Molecular methods are now the gold standard for identifying *Francisella* sp. strains at the species, subspecies, and even genotype levels [20,21]. Real-time PCR tests are more adapted for the rapid and accurate detection and identification of *F. tularensis*. However, these tests are not used on a routine basis in many diagnostic laboratories and remain costly. MALDI TOF (matrix-assisted laser desorption ionization-time of flight) mass spectrometry (ms) is now routinely used in most clinical microbiology laboratories for the rapid and accurate identification of most human pathogens [22,23,24]. However, the currently available diagnostic databases contain few or no strains of *F. tularensis*. Therefore, the MALDI TOF ms method has been poorly evaluated for the identification of *Francisella* species.

In this study, we developed and standardized a procedure for the MALDI TOF ms identification of *F. tularensis*, including optimizing culture in a biosafety level 3 laboratory, bacterial inactivation of this highly infectious pathogen, and MALDI TOF ms analysis procedure. We built a specific *F. tularensis* database that could be used to rapidly identify this bacterium, especially in the context of the expertise carried out by our reference laboratory for tularemia diagnosis.

## 2. Materials and Methods

### 2.1. Bacterial Strains

We tested 68 clinical strains and the live vaccine strain (LVS) of *Francisella tularensis* subsp. *holarctica*, as well as *F. tularensis* subsp. *novicida* U112 and *F. philomiragia* ATCC 25015 as reference strains. Clinical strains of *F. tularensis* are owned by the French National Reference Center for tularemia (FNRCT), which holds specific authorizations from the Agence Nationale de Sécurité du Médicament et des Produits de Santé (ANSM, authorization numbers ADE-103892019–7 and AMO-103902019–9). These bacterial strains were identified at the subspecies level through 16S rRNA gene sequencing and a type B-specific real-time PCR test [25].

To assess the specificity of the *Francisella* database that we built during this study, we tested a panel of 165 strains from 63 bacterial species commonly isolated in a clinical microbiology laboratory, including 26 Gram-positive and 37 Gram-negative species. This panel included 32 strains belonging to the small Gram-negative HACEK group of bacteria (*Haemophilus*, *Aggregatibacter*, *Capnocytophaga*, *Eikenella*, and *Kingella*) and the reference strain *Brucella melitensis* 16M reference (ATCC 23456) (Table 1). All these bacterial strains (except *B. melitensis* 16M) were isolated and identified in the Grenoble Alpes University Hospital’s bacteriology laboratory, using the MALDI Biotyper for In Vitro Diagnostic (MBT IVD) database of the Biotyper Microflex LT/SH Maldi-MS System system from Bruker Daltonics (Wissembourg, France).

### 2.2. Bacterial Cultures

The *Francisella* sp. cultures were carried out in a level 3 biological safety laboratory (BSL3) on chocolate agar plates supplemented with PolyViteX^®^ (Choco-PVX, bioMérieux, Lyon, France). The LVS strain of *F. tularensis* was grown under different culture conditions to evaluate the optimal parameters to carry out MALDI TOF ms analyses. We tested the combinations of the following parameters: an incubation time of cultures of 24 h, 48 h, 72 h, or 96 h; an incubation temperature of 30 °C or 37 °C; incubation in aerobic, 5% CO2-enriched, microaerophilic, or anaerobic atmospheres. We also tested successive subcultures of the LVS strain on Choco-PVX incubated at 37 °C, in aerobiosis, to evaluate the evolution of the MALDI TOF ms spectra over time. The optimal conditions for MALDI TOF ms analysis of the LVS strain were then applied to all other *Francisella* sp. strains and to control strains that did not belong to the *Francisella* genus.

### 2.3. Bacterial Inactivation and Protein Extraction

Inactivation of *Francisella* strain before MALDI TOF ms analysis was evaluated using the LVS strain, in the BSL3 laboratory. One or two bacterial colonies obtained on Choco-PVX agar after 48-h incubation were resuspended in 300 µL of ultra-pure water, which corresponded to a bacterial inoculum of 3 to 5 × 10^6^ CFU/mL, as determined by CFU counts. Then, 900 μL of absolute ethanol was added to obtain a final ethanol concentration of 75%. This suspension was incubated for 10 min at room temperature. It was then centrifuged at 13,000 rpm for 5 min to obtain a bacterial pellet. After removing the supernatant, the bacterial pellet was resuspended in 25 µL of 70% formic acid and 25 µL of 100% acetonitrile. After further centrifugation (13,000 rpm for 5 min), the supernatant containing the extracted bacterial proteins was recovered and analyzed using MALDI TOF ms. 

Assessment of the bacterial viability was performed before the inactivation protocol (growth control), after exposure of bacteria to 75% ethanol, and, finally, at the end of the protein extraction protocol. This procedure allowed us to assess the efficacy of the inactivation protocol at intermediate and final steps. At each time, 10 µL of the bacterial suspension was inoculated to Choco-PVX agars, which were incubated at 37 °C, in 5% CO2-enriched atmosphere, for ten days to check the formation of bacterial colonies.

### 2.4. MALDI TOF ms Procedure

MALDI TOF ms analyses were performed using the Biotyper Microflex LT/SH Maldi-MS System from Bruker Daltonics (Wissembourg, France). For each bacterial strain tested, the supernatant containing the extracted proteins (as described in the previous section) was deposited (1 µL, in triplicate) on a reusable polished steel target and left to dry. The same procedure was used for a bacterial test standard (BTS), which allowed the calibration of the mass spectrometer before each batch of tests. Then, one μl of α-cyano-4-hydroxycinnamic acid (HCCA) matrix was added above the dried supernatants. The plate was then introduced into the mass spectrometer for analysis after the matrix HCCA had dried. 

The mass spectra were analyzed using the flexAnalysis^®^ software from Bruker Daltonics (Wissembourg, France) and the Bruker BioTyper database for in vitro diagnosis (MBT IVD, Library 9.0), containing 8326 main spectra profiles (MSPs) corresponding to most bacterial species routinely isolated from humans in a clinical microbiology laboratory. The MALDI TOF ms analysis gives a species identification associated with a score value (range 0 to 3) that indicates the degree of its reliability. A score between 2 and 3 is considered a reliable identification at the species level; a score between 1.99 and 1.70 is identification with low confidence at the species level but probable at the genus level; a score below 1.69 does not give any reliable species identification.

### 2.5. MALDI TOF ms Francisella Database Building

Main spectra profiles (MSPs) were determined for 24 clinical strains of *Francisella tularensis* subsp. *holarctica*, the vaccine strain LVS, *F. tularensis* subsp. *novicida* U112, and *F. philomiragia* ATCC 25015. For the creation of these MSPs, we used the protocol recommended by Bruker Daltonics. For each strain, one μL of the protein extract was deposited eight times on different metal target plate positions to assess the reproducibility of the results. The BTS was used for calibration of the mass spectrometer. The following steps and ms analyses were the same as described above.

For each bacterial strain, the eight spots were analyzed three times, giving 24 spectra. These spectra were then processed using the flexAnalysis^®^ software to create MSPs. This software was used to eliminate spectra with “ghost peaks” or “aberrant peaks” or those with a flat baseline. We also checked that, for each mass peak, the difference between the lowest and the highest detected masses was lower than 500 ppm. Spectra exceeding this difference were removed. The final database (referred to as the FNRCT database) consisted of 24 MSPs from each *Francisella* sp. tested.

### 2.6. MALDI TOF ms Francisella Database Evaluation

The analytical sensitivity of the FNRCT database was evaluated by testing 69 *Francisella tularensis* subsp. *holarctica* strains (including 68 clinical strains and the LVS strain). Its specificity was assessed by testing *F. tularensis* subsp. *novicida* U112, *F. philomiragia* ATCC 25015, and 165 bacterial strains belonging to 63 species other than *Francisella* sp. All these bacterial strains were tested against the BioTyper MBT IVD and FNRCT databases.

## 3. Results

### 3.1. F. tularensis LVS Strain Inactivation and MALDI TOF ms Analysis

CFU counts determined before any inactivation process confirmed the viability of the LVS strain. After 10-min exposure to 75% ethanol at room temperature, no bacterial growth was observed despite ten days incubation of the inoculated Choco-PVX agars. The same result was obtained after exposure of bacterial suspensions to 75% ethanol, 70% formic acid, and acetonitrile, which corresponded to the entire process usually recommended by Bruker Daltonics ^®^ (Wissembourg, France) for the inactivation of bacteria before MALDI TOF ms analysis.

### 3.2. F. tularensis LVS Culture Optimization

**Figure 1:**
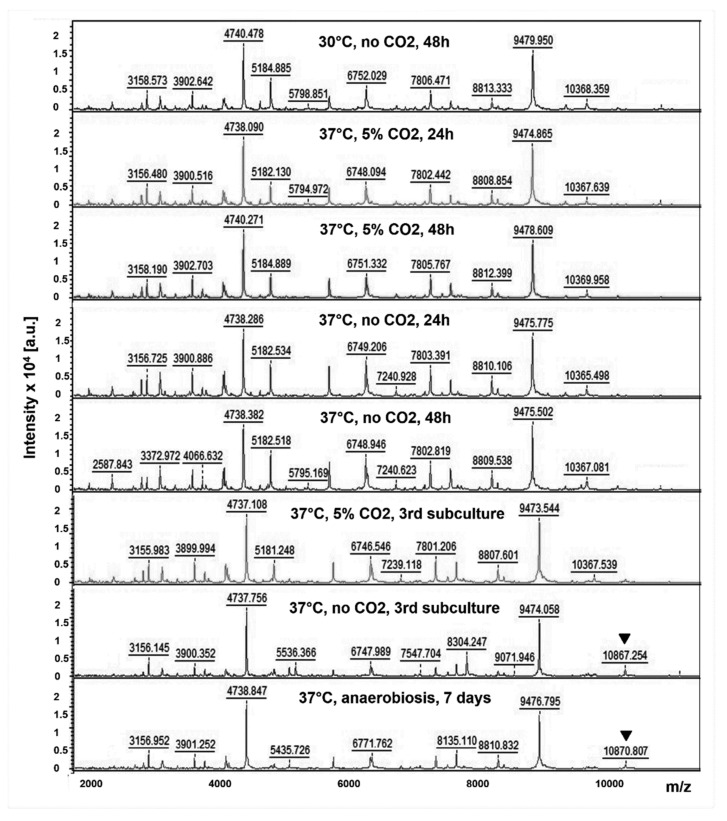
MALDI TOF mass spectra obtained with the LVS strain of *F. tularensis* subsp. *holarctica*, at different culture conditions: 30 °C or 37 °C, with or without 5% CO2, with an incubation time of 24 h or 48 h, and after one subculture (unspecified) or three subcultures. A mass peak in the range 10,360–10,370 Da was missing and replaced by a new peak in the range 10,860–10,870 Da (arrows) when the LVS strain was grown in the anaerobic atmosphere for ten days or after three or more subcultures at 37 °C, without CO2.

Optimal growth of *F. tularensis* LVS was obtained at 37 °C, after 48 h incubation of Choco-PVX media, with or without 5% CO2. Large colonies (2–3 mm) allowed easy preparation of a bacterial suspension from two to three colonies for reliable MALDI TOF ms analysis. In the same condition, smaller colonies (1–2 mm in diameter) were visible after 24-h incubation of the plates. Bacterial growth was slower at 30 °C, with or without CO2. When performing four subcultures (every 24 h), at 37 °C or 30 °C, larger colonies were progressively obtained. The LVS strain did not grow in a strict anaerobic atmosphere after 72-h incubation of the Choco-PVX plates at 30 °C or 37 °C. In contrast, bacterial growth was detected after ten days of incubation of cultures. In a microaerophilic atmosphere, bacterial colonies were detected after 72 h of incubation, either at 30 °C or 37 °C (Figure 1). Whatever the incubation conditions, the LVS colonies were convex, opaque, shiny, and cream-colored (Figure 1). To standardize and simplify the culture procedure before MALDI TOF ms analysis, we considered incubation of Choco-PVX plates at 37 °C, for 48 h, in 5% CO2-enriched atmosphere as optimized conditions for the LVS strain. The same parameters were then applied to all other bacterial strains tested in this study.

### 3.3. F. tularensis LVS MALDI TOF Mass Spectra According to Culture Conditions

We assessed the influence of the culture conditions of *F. tularensis* LVS strains on MALDI TOF mass spectra. Very similar spectra were obtained when the LVS strain was grown on Choco-PVX at 30 °C or 37 °C, with or without CO2, and for 24 h up to 96 h. The same spectra were also obtained in microaerophilic conditions at 30 °C or 37 °C. Up to four subcultures (one every 24 h) of the LVS strain on Choco-PVX agar, incubated at 37 °C, with or without CO2, did not change the mass spectra profiles. In all these conditions, MALDI TOF mass spectra were composed of 16 striking peaks (Figure 1). The two major peaks were located at mass values between 4736 Da and 4740 Da and between 9472 Da and 9480 Da. In contrast, a mass peak in the range of 10,360–10,370 Da was missing and replaced by a new peak in the range 10,860–10,870 Da when the LVS strain was grown in the anaerobic atmosphere for ten days or after three or more subcultures at 37 °C, without CO2. (Figure 1).

### 3.4. Identification of Francisella Strains Using the MBT IVD Database

*F. philomiragia* identification was reliable (score of 2.48) using the MBT IVD database, although this one contains a single strain of this species. *F. tularensis* subsp. *novicida* was identified as *Comamonas kerstersii* (score, 1.31). The LVS strain was identified as *Lactobacillus graminis* (score, 1.32). The following species identifications were obtained for strains of *F. tularensis* subsp. *holarctica*, with scores ranging from 1.24 to 1.53: *Streptococcus oralis*, *S. uberis*, *S. gallolyticus*, *Lactococcus piscium*, *Lactobacillus coryniformis*, *L. curvatus*, *Arthrobacter russicus*, and *Klebsiella pneumonia* subsp. *ozaenae*. *Lactococcus piscium* and *Lactobacillus coryniformis* were the most frequently proposed identifications.

### 3.5. Analytical Sensitivity Evaluation of the FNRCT Database

The FNRCT database finally contained 27 *Francisella* strains, including 24 clinical strains of *F. tularensis* subsp *holarctica*, the LVS strain of the same subspecies, and the reference strains *F. tularensis* subsp. *novicida* U112 and *F. philomiragia* ATCC 25015. For each strain, 24 spectra were available. As for *F. tularensis subsp. holarctica*, a few variations in the intensity of mass peaks were noted between strains. Hence, when one of the 24 strains used to build the FNRCT database was tested with this database, this specific strain was proposed first, then the other strains of the same subspecies. Therefore, the flexAnalysis^®^ software was able to differentiate the spectral profiles of the 24 clinical strains of *F. tularensis* subsp. *holarctica* constituting this base.

The 68 clinical strains and the LVS strain of *Francisella tularensis* subsp. *holarctica* were identified with the FNRCT database at the species level. A score of 3 was obtained for the *F. tularensis* subsp. *holarctica* strains used to build the FNRCT database. For all other strains of this subspecies, scores ranged between 2.7 and 3, which corresponded to a reliable identification.

*F. tularensis* subsp. *novicida* U112 and *F. philomiragia* ATCC 25015 were accurately identified using the FNRCT database, with scores of 3 in both cases. *F. tularensis* susp. *holarctica* was proposed with a score of 1.83 (identification of low confidence) for *F. tularensis* subsp. *novicida* and 1.01 (unreliable) for *F. philomiragia*. Therefore, these species and subspecies could be accurately differentiated.

### 3.6. Analytical Specificity Evaluation of the FNRCT Database

Among the most challenging small, Gram-negative bacteria belonging to the “HACEK” group and *Brucella* species, the scores were higher than 2 when using the MBT IVD database (Table 2). In contrast, the identification scores of these species, compared to each other, were always less than 2. Therefore, these strains could be accurately identified and differentiated using the MBT IVD database. Using the FNRCT database, the above species displayed scores ranging from 0.19 to 1.2 (i.e., unreliable identification) (Table 2).

The other bacterial species commonly isolated in routine diagnostic laboratories displayed scores greater than 2 with the MBT IVD database. For each of these species, a score of less than 2 was proposed for the most closely related species, allowing easy differentiation of species. However, some phylogenetically closely related species could not be differentiated, such as *Neisseria flavescens* with *N. subflava*, as well as *Streptococcus dysgalactiae* and *S. pyogenes*. When tested using the FNRCT database, the above strains gave identification scores of less than 1.69 (unreliable identification) (Table 3). *F. tularensis* subsp. *holarctica*, *F. tularensis* subsp. *novicida*, *F. philomiragia*, or several of these *Francisella* species were the proposed identifications for these strains. However, identification scores ranged from 0.14 to 1.13 for *F. tularensis* subsp. *holarctica*, 0.13 to 1.06 for *F. tularensis* subsp. *novicida*, and 0.13 to 0.60 for *F. philomiragia*.

## 4. Discussion

Our main objective was to develop a database for the rapid and accurate identification of *F. tularensis* subsp. *holarctica* strains, using the Biotyper Microflex LT/SH Maldi-MS System (Bruker Daltonics). As a national reference center for tularemia, this would allow us to perform rapid analysis of bacterial strains sent to our laboratory for confirmation of the identification of this major human pathogen. Identification of *F. tularensis* subsp. *holarctica* within a few minutes of reception would allow the implementation of optimized prophylactic and therapeutic interventions. Treatment of the infected patient could be adapted more rapidly. Besides this, adapted prevention measures would reduce the risk of contamination of the laboratory personnel handling cultures of this pathogen. It should be stressed that molecular tests will still be needed to determine the *F. tularensis* subspecies and genotype involved.

We first defined the optimal conditions for *F. tularensis* growth to obtain high quality and reproducible MALDI TOF mass spectra. *F. tularensis* is considered a strictly aerobic bacterium, with optimum growth at 37 °C, in cysteine-enriched media. Bacterial colonies are usually obtained within 2 to 4 days for primary isolation and 24–48 h after subculture. The LVS strain of *F. tularensis* subsp. *holarctica* was used to perform these experiments because of its low virulence. This strain was cultivated on chocolate agar supplemented with PolyViteX^®^ (Choco-PVX, bioMérieux), a medium that contains a nutritive base enriched with factors X (hemin) and V (nicotinamide adenine dinucleotide), supplied by hemoglobin, and a multi-vitamin supplement (PolyViteX^®^). Using Choco-PVX, optimum growth was obtained at 37 °C, within 24–48 h, in the presence or absence of 5% carbon dioxide. The LVS strain also grew well (although slowly) at 30 °C without CO2, and large colonies were obtained after 48-h incubation of the Choco-PVX plates. Surprisingly, LVS bacterial colonies were detected after 72 h of incubation in the microaerophilic atmosphere, both at 30 °C and 37 °C. The growth of *F. tularensis* in the microaerophilic atmosphere might be related to this bacterium’s ability to multiply in the oxygen-poor environment of the phagocytic cells. No growth was observed after three days of incubation in an anaerobic atmosphere, whereas the growth observed in such conditions after ten-day incubation of these cultures likely reflects the loss of a strict anaerobic condition over time. After several subcultures, large colonies of *F. tularensis* LVS were obtained more rapidly, but this procedure was not adapted for the rapid identification of this pathogen.

We demonstrated that an easy and rapid sample preparation before MALDI TOF ms analysis allowed reliable inactivation of *F. tularensis*, a highly infectious pathogen through the respiratory route. Although 70% of ethanol may not be active enough to inactivate spore-forming bacteria (*Bacillus* and *Clostridium* species) [26], it completely inactivated a high inoculum of *F. tularensis*. This first step of our protein extraction procedure could be performed in a biosafety level 2 laboratory under a biosafety cabinet for type B strains. The cultures of the more virulent type A strains should preferably be handled in a biosafety level 3 laboratory. Although the ethanol–formic acid extraction method is not mandatory for Gram-negative bacteria without a large glycocalyx or capsule layer, such as *F. tularensis*, we used it to obtain high-quality spectra. The MALDI TOF mass spectra obtained with the LVS strain were almost identical, whatever the incubation conditions. The flexAnalysis^®^ software allowed us to differentiate 16 different peaks with mass values ranging from approximately 2000 Da up to 11,000 Da. Only for mass spectra obtained after ten-days incubation in anaerobic conditions, or after three or more subcultures at 37 °C, without CO2, a mass peak at 10,360–10,370 Da was missing and replaced by a new peak at 10,860–10,870 Da. In these two conditions, prolonged incubation of cultures likely induced significant changes in the MALDI TOF mass spectra. To standardize and simplify the culture procedure before MALDI TOF ms analysis, we selected an incubation of Choco-PVX plates at 37 °C, for 48 h, in 5% CO2-enriched atmosphere as optimized conditions for *Francisella* species but also for other species including fastidious growth human pathogens. In this study, we did not evaluate the minimum bacterial load needed to obtain high-quality mass spectra. However, as few as one or two bacterial colonies were enough to obtain highly reproducible mass spectra and reliable identification scores. Considering that protein extracts (50 µL final volume) were obtained from approximately 10^6^ CFUs, and 1µL of this suspension was analyzed by MALDI TOF ms, it can be assumed that the protein content from approximately 20–30 × 10^3^ bacteria was enough for accurate *F. tularensis* identification.

The FNRCT owns a collection of 68 clinical strains of *Francisella tularensis* subsp. *holarctica*, which was tested during this study. Most of the clinical strains were isolated from patients with different clinical forms of tularemia, infected in France between 2004 and 2016. These infections were sporadic and unrelated. All these strains showed multiplication capacities similar to each other and to that of the LVS strain. We used 25 *F. tularensis* strains (including 24 clinical strains and the LVS strain) and the reference strains *F. tularensis* subsp. *novicida* U112 and *F. philomiragia* ATCC 25015 to build an *F. tularensis* specific MALDI TOF ms database (referred to as the FNRCT database). This database was then validated by testing a total of 68 clinical strains of *F. tularensis* subsp. *holarctica* (including the 24 used for database building). All these strains were accurately identified at the species level, with identification scores between 2.7 and 3. Besides this, subtle differences in mass spectra between these strains allowed the fexAnalysis^®^ software from Bruker Daltonics (Wissembourg, France) to differentiate between the 24 *F. tularensis* strains used for FNRCT database development. This result suggested that specific signatures may potentially allow assessment of the epidemiological link between several *F. tularensis* strains, although this remains to be established. Whole-genome sequencing with SNP analyses is the current gold standard for molecular epidemiology evaluation of *F. tularensis* infections [27].

Interestingly, *F. tularensis* subsp. *novicida* could be differentiated from *F. tularensis* susbp. *holarctica* strains, although the two subspecies have similar genome sequences and an average nucleotide identity higher than 98% [20,21]. This finding is of tremendous importance because both microorganisms may be isolated from human samples, while only *F. tularensis* susbp. *holarctica* is an etiological agent of tularemia. *F. tularensis* subsp. *novicida* is an aquatic bacterium that has been rarely associated with human infections, especially pneumonia [6]. *F. philomiragia*, another aquatic bacterium occasionally involved in human infections [28], was also readily identified and differentiated from the two *F. tularensis* subspecies tested. It should be noted that, at the time of this study, the Bruker Daltonics (Wissembourg, France) MBT IVD database only contained a single strain of *F. philomiragia* and no *F. tularensis* strain Thus, it is only able to identify this *Francisella* species. 

To assess the FNRCT database’s specificity, we tested 165 strains from 63 bacterial species commonly isolated in a clinical microbiology laboratory. They included 26 Gram-positive and 37 Gram-negative species. These strains were readily identified using the MBT IVD database of the Biotyper Microflex^®^ system from Bruker Daltonics. When tested against the FNRCF database, all these strains displayed identification scores of less than 1.69 (unreliable identification). More specifically, 32 strains belonging to the small Gram-negative HACEK group of bacteria and a reference strain of *Brucella melitensis* were investigated both because, as for *F. tularensis*, they appear as small Gram-negative bacteria on Gram stain and they may be isolated from blood cultures (especially from endocarditis patients). Moreover, *F. tularensis* subsp. *holarctica* and *Brucella* sp. are associated with a high risk of laboratory infection [15,29]. It is, therefore, essential to rapidly differentiate the HACEK, *Brucella*, and *F. tularensis* species. Using the MBT IVD database, all the HACEK species could be accurately identified and differentiated, with an identification score higher than 2. Previous studies have stressed the usefulness of MALDI TOF ms for reliable identification of these species [30]. When tested against the FNRCT database, the HACEK and *Brucella* species displayed scores below 1.3 (unreliable identification). Therefore, by testing the ms profile obtained for a given bacterial strain against the MBT IVD and FNRCT databases, accurate and rapid identification of the HACEK, *Brucella*, and *F. tularensis* species could be obtained.

Rudrik et al. [17] reported low-level identification scores (<1.4) when five *F. tularensis* strains (tularemia agents including the LVS) were tested in six different laboratories, using the IVD or SR libraries of the Bruker MALDI Biotyper (Bruker Daltonics). An identification rate of 24.4% of *F. tularensis* was obtained in three laboratories using the RUO library of the bioMerieux Vitek MS library (bioMérieux). However, with this system, 55.6% of the protein extracts obtained from *F. tularensis* subsp. *novicida* (also referred to as *F. novicida*) were misidentified as *F. tularensis*. A study by Karatuna et al. [31], in Turkey, evaluated a large number (*n* = 75) of clinical isolates of *F. tularensis* subsp. *holarctica*. All tested strains were identified as *Francisella* sp. using the Security-Relevant (SR) database of the Bruker MALDI Biotyper. However, only 19/75 (25.3%) strains were identified at the species level (score > 2), while 56/75 (74.7%) were identified at the genus level (score 1.7 and 2). All these strains were confirmed to belong to the *F. tularensis* subsp. *holarctica* subspecies by the positivity of PCR amplification of a specific DNA sequence of the region of difference 1 (RD1) [25]. Interestingly, identification scores obtained with the type A SCHU S4 strain of *F. tularensis* were always lower than those of the LVS type B strain. Seibold et al. [32] reported the usefulness of MALDI TOF ms to identify 50 strains belonging to the genus *Francisella*, taking the 23 S rRNA gene sequence as the reference standard. These strains belonged to the four subspecies of *F. tularensis* or *F. philomiragia*. Accurate identification scores (2.33 to 2.56) were obtained using the Microflex LT and Ultraflex III TOF/TOF mass spectrometers from Bruker Daltonics.

Lundquist et al. [33] reported the usefulness of surface-enhanced laser desorption/ionization time-of-flight (SELDI-TOF) technology to discriminate *F. tularensis* subspecies (i.e., subsp. *tularensis*, *holarctica*, *mediasiatica*, and *novicida*). SELDI-TOF ms allows the selective absorption of proteins on the chromatographic array surface and thus the direct analysis of bacterial lysates. The homogeneous binding of target proteins allows improved reproducibility of ms analyses. Therefore, both mass-to-charge (m/z) ratio but also intensity values can be taken into account. Lundquist et al. [33] tested 16 strains belonging to one of the four subspecies of *F. tularensis*, using the PBS IIC instrument (Ciphergen Biosystems, CA, USA). All subspecies could be accurately identified and differentiated. The authors also emphasized that a SELDI TOF ms approach could also help to detect functional differences between bacterial strains of individual proteins. Seibold et al. [34] also reported the usefulness of SELDI-TOF ms to differentiate the four subspecies of *F. tularensis*, both among one another and with *F. philomiragia*. The main limitation of this technology is that this costly equipment is not available in most clinical microbiology laboratories.

A limitation of our study was the lack of evaluation of our FNRCT database’s ability to identify type A *F. tularensis* strains because these were not available in our laboratory. More generally, none of the published studies evaluating the usefulness of mass spectrometry for rapid identification of *Francisella* sp. have taken into account the many species of this genus newly characterized in the last decade. However, some of them have been occasionally isolated from humans [35].

## 5. Conclusions

In conclusion, we developed an easy and rapid procedure for identifying *F. tularensis* subsp. *holarctica* using MALDI TOF mass spectrometry. This instrumentation is now available in most clinical microbiology laboratories. Moreover, bacterial strains sent to our FNRCT for expertise have usually been grown on Choco-PVX plates, which are rich media widely used for fastidious growth and difficult-to-identify bacteria. We have shown that incubation of Choco-PVX at 30 °C or 37 °C, in aerobic or 5% CO2-enriched atmosphere, for less than 72 h, allows accurate identification of *F. tularensis* subsp. *holarctica* using our FNRCT database and the Biotyper Microflex system from Bruker Daltonics. Therefore, identification of this subspecies can be readily achieved in our laboratory within minutes of the reception of the bacterial culture. Alternatively, a Choco-PVX culture of the bacterial strain to be identified will be required, and *F. tularensis* subsp. *holarctica* identification will be obtained within 48 h. Our database only contains type B strains of *F. tularensis*, the only subspecies found in Europe, but could likely identify (but may not differentiate) type A strains. The FNRCT database also allowed accurate identification and differentiation of the closely related species *F. tularensis* subsp. *novicida* and *F. philomiragia*, which are currently the *Francisella* species that are most likely to be isolated from human samples in routine clinical microbiology practice. Rapid and accurate identification of *F. tularensis* subsp. *holarctica* has many consequences, including better management of the infected patients, more rapid detection of unusual situations (e.g., an outbreak that could lead to suspicion of intentional release of this pathogen), and prevention of laboratory infections. Besides this, sharing the ms database between laboratories using the same equipment could optimize tularemia surveillance in humans and animals.

## Figures and Tables

**Table 1:** Bacterial strains used in this study.

Species	Species
*Francisella tularensis* subsp. *holarctica* (68 clinical strains)	
*Francisella tularensis* subsp. *holarctica* LVS	*Haemophilus influenzae*
*F. novicida* U112	*Haemophilus parainfluenzae*
*F. philomiragia* ATCC 25015	*Hafnia alvei*
*Acidovorax temperans*	*Kingella kingae*
*Acinetobacter baumannii*	*Klebsiella pneumoniae*
*Acinetobacter pittii*	*Klebsiella variicola*
*Actinomyces odontolyticus*	*Lactobacillus rhamnosus*
*Aggregatibacter actinomycetemcomitans*	*Morganella morganii*
*Aggregatibacter aphrophilus*	*Neisseria subflava*
*Bacillus subtilis*	*Neisseria flavescens*
*Bacteroides fragilis*	*Paenibacillus amylolyticus*
*Bacteroides ovatus*	*Pediococcus acidilactici*
*Bacteroides thetaiotaomicron*	*Propionibacterium acnes*
*Bacteroides vulgatus*	*Proteus mirabilis*
*Brucella melitensis*	*Pseudomonas aeruginosa*
*Campylobacter jejuni*	*Serratia ureilytica*
*Capnocytophaga* sp.	*Sphingomonas paucimobilis*
*Citrobacter amalonaticus*	*Sphingomonas sp*
*Citrobacter braakii*	*Staphylococcus aureus*
*Citrobacter freundii*	*Staphylococcus epidermidis*
*Citrobacter koseri*	*Staphylococcus haemolyticus*
*Citrobacter sedlakii*	*Staphylococcus hominis*
*Corynebacterium simulans*	*Staphylococcus lugdunensis*
*Eikenella corrodens*	*Staphylococcus pasteuri*
*Enterobacter aerogenes*	*Staphylococcus warneri*
*Enterobacter asburiae*	*Streptococcus agalactiae*
*Enterobacter cloacae*	*Streptococcus constellatus*
*Enterococcus casseliflavus*	*Streptococcus gallolyticus*
*Enterococcus faecalis*	*Streptococcus pyogenes*
*Enterococcus faecium*	*Streptococcus mitis*
*Escherichia coli*	*Streptococcus oralis*
*Gemella haemolysans*	*Turicella otitidis*

**Table 2:** Identification scores obtained with *Haemophilus*, *Aggregatibacter*, *Capnocytophaga*, *Eikenella*, and *Kingella* (HACEK group of species) and *Brucella melitensis* using the MBT IVD or FNRCT databases.

Species (Strain)	MBT IVD Score	MBT IVD 2nd Proposal Score	FNRCT Database Score
*Haemophilus influenzae*	2.44	NA	*F. tul. novicida*, 0.79
*Haemophilus influenzae*	2.3	NA	*F. tul. holarctica*, 0.49
*H. parainfluenzae* (1)	2.51	*H. parainfluenzae*, 2.33	*F. tul. holarctica*, 0.88
*H. parainfluenzae* (2)	2.49	*Pasteurella canis*, 1.64	*F. tul. holarctica*, 0.9
*H. parainfluenzae* (3)	2.28	*H. haemolyticus*, 1.6	*F. philomiragia*, 0.74
*H. parainfluenzae* (4)	2.2	*H. haemolyticus*, 1.67	*F. tul. holarctica*, 0.7
*H. parainfluenzae* (5)	2.01	*Neisseria gonorrheae*, 1.39	*F. philomiragia*, 0.65
*A. actinomycetemcomitans* (1)	2.36	*Sphingomonas yabuuchiae*, 1.42	*F. tul. holarctica*, 0.72
*A. actinomycetemcomitans* (2)	2.27	*Sphingomonas aquatilis*, 1.57	*F. tul. holarctica*, 0.69
*A. actinomycetemcomitans* (3)	2.44	*H. parainfluenzae*, 1.41	*F. philomiragia*, 0.19
*A. actinomycetemcomitans* (4)	2.41	*A. aphrophilus*, 1.45	*F. philomiragia*, 0.6
*A. actinomycetemcomitans* (5)	2.39	*H. influenza*, 1.35	*F. tul. holarctica*, 0.7
*A. aphrophilus* (1)	2.37	*H. haemolyticus*, 1.83	*F. tul. holarctica*, 0.32
*A. aphrophilus* (2)	2.23	*H. haemolyticus*, 1.64	*F. philomiragia*, 0.51
*A. aphrophilus* (3)	2.08	*H. haemolyticus*, 1.68	*F. philomiragia*, 0.81
*A. aphrophilus* (4)	2.05	*A. actinomycetemcomitans*, 1.47	*F. tul. holarctica*, 0.7
*A. aphrophilus* (5)	2.02	*Streptococcus intermedius*, 1.61	*F. tul. holarctica*, 0.71
*Capnocytophaga* sp. (1)	2.21	*Hafnia alvei*, 1.35	*F. tul. holarctica*, 0.4
*Capnocytophaga* sp. (2)	2.09	*Agromyces rhizospherae*, 1.35	*F. tul. holarctica*, 0.68
*Capnocytophaga* sp. (3)	2.01	*Pseudomonas asplenii*, 1.39	*F. tul. holarctica*, 0.83
*Capnocytophaga* sp. (4)	1.97	*Branhamella catarrhalis*, 1.39	*F. tul. holarctica*, 0.55
*C. sputigena*	2.49	*Capnocytophaga ochracea*, 1.81	*F. tul. novicida*, 0.22
*E. corrodens* (1)	2.29	*Burkholderia anthina*, 1.51	*F. tul. holarctica*, 0.62
*E. corrodens* (2)	2.27	*Burkholderia anthina*, 1.37	*F. tul. holarctica*, 0.4
*E. corrodens* (3)	2.13	*Burkholderia cepacia*, 1.41	*F. tul. novicida*, 0.39
*E. corrodens* (4)	2.09	*Neisseria meningitides*, 1.41	LVS, 0.33
*E. corrodens* (5)	2.01	*Pseudomonas koreensis*, 1.38	LVS, 0.52
*K. kingae* (1)	2.6	*Kingella kingae*, 2.43	*F. tul. holarctica*, 0.89
*K. kingae* (2)	2.46	*Jonesia denitrificans*, 1.31	*F. tul. holarctica*, 1.2
*K. kingae* (3)	2.6	*Kingella kingae*, 2.43	*F. tul. holarctica*, 1.05
*K. kingae* (4)	2.45	*Pseudomonas monteilii*, 1.41	*F. tul. holarctica*, 0.9
*K. kingae* (5)	2.5	*Kingella kingae*, 2.5	*F. tul. holarctica*, 0.86
*B. melitensis* M16	2.38	*Clostridium botulinum*, 0.97, or *Bacillus anthracis* (0.91–1.09)	*F. philomiragia*, 0.31–0.59

A score between 2 and 3: reliable species identification; 1.99 to 1.70: identification only probable at the genus level; < 1.69: no reliable species identification.

**Table 3:** Identification scores obtained with bacterial species other than *Francisella* sp. or the HACEK group, using the MBT IVD or FNRCT databases.

Species (Number of Strains)	MBT IVD Database Score	Database Score Against One or Several of the Following *Francisella* Species		
		*F. tul.* *holactia*	*F. tul.* *novicida*	*F. philomiragia*
*Acidovorax temperans*	2.23	0.47		
*Acinetobacter baumannii*	2.35	0.16		
*Acinetobacter pittii*	2.3		0.52	
*Actinomyces odontolyticus*	2.05	0.63		
*Enterobacter cloacae*	2.31	0.40		
*Bacteroides fragilis* (2)	2.33	0.14	0.41	
*Bacteroides ovatus*	2.17	0.17		
*Bacillus subtilis*	1.93		1.06	
*Bacteroides thetaiotaomicron*	2.35	0.32		
*Bacteroides vulgatus*	2.62	0.33		
*Campylobacter jejuni*	2.11			
*Citrobacter amalonaticus*	2.31	0.74		
*Citrobacter braakii*	2.17	0.83		
*Citrobacter freundii* (4)	2.07–2.43	0.71–0.98		
*Citrobacter koseri*	2.39	0.25		
*Citrobacter sedlakii*	2.41	0.69		
*Corynebacterium simulans*	2.08	0.37		
*Enterobacter aerogenes*	2.25	1.01–1.11		
*Enterobacter asburiae*	2.38	0.49		
*Enterobacter cloacae* (4)	2.29–2.41	0.35–0.59		0.13–0.5
*Enterococcus casseliflavus*	2.16–2.22	0.69–0.91		
*Enterococcus faecalis* (5)	2.34–2.41	0.6		
*Enterococcus faecium* (2)	2.42	0.58	0.49	
*Gemella haemolysans*	2.06	0.55		
*Escherichia coli* (7)	2.36–2.55	0.56–0.78		
*Hafnia alvei* (6)	2.26–2.6	0.37–0.72	0.76	
*klebsiella pneumoniae* (9)	2.16–2.54	0.43–0.92		
*Klebsiella variicola*	2.48	0.74		
*Lactobacillus rhamnosus*	2.38	0.70		
*Morganella morganii* (8)	2.42–2.6	0.57–0.95		
*Neisseria flavescens*	2.14		0.25	
*Neisseria subflava*	2.7	0.27		
*Paenibacillus amylolyticus*	2.14			0.56
*Pediococcus acidilactici*	2.25		0.65	
*Propionibacterium acnes*	2.47			0.39
*Proteus mirabilis* (3)	2.4–2.46	0.86–1.07		
*Pseudomonas aeruginosa* (10)	2.05–2.51	0.22–0.46	0.13–0.76	0.47–0.60
*Serratia ureilytica*	2.32	0.47		
*Sphingomonas paucimobilis*	2.48	1.03		
*Staphylococcus aureus* (12)	2.26–2.5	0.27–0.60		0.33–0.6
*Staphylococcus epidermidis* (13)	2.04–2.23	0.1–0.7		
*Staphylococcus haemolyticus* (2)	2.13–2.33	0.58–0.59		
*Staphylococcus hominis*	2.21	0.54		
*Staphylococcus lugdunensis* (3)	2.06–2.25	0.45	0.74	
*Staphylococcus pasteuri*	2.12			0.47
*Staphylococcus warneri* (2)	2.09–2.1	0.54–0.73		
*Streptococcus agalactiae*	2.35		0.34	
*Streptococcus anginosus*	2.06	0.90		
*Streptococcus constellatus* (2)	2.13–2.24	1.11–1.13		
*Streptococcus gallolyticus*	2.25	0.73		
*Streptococcus mitis*	2.01	0.92		
*Streptococcus oralis*	2.14	0.68		
*Streptococcus pyogenes* (2)	2.42–2.44	0.78–0.98		
*Turicella otitidis*	2.07		0.35	

A score between 2 and 3: reliable species identification; 1.99 to 1.70: identification only probable at the genus level; < 1.69: no reliable species identification.
